# Incidence, Patient-Related Risk Factors, and Outcomes of Postoperative Pneumonia after Cholecystectomy: A Population-Based Cohort Study

**DOI:** 10.1155/2021/6614885

**Published:** 2021-05-10

**Authors:** Yun-Hui Teng, Fu-Chao Liu, Keng-Hao Liu, Jr-Rung Lin, Huang-Ping Yu

**Affiliations:** ^1^Department of Anesthesiology, Chang Gung Memorial Hospital, Taoyuan, Taiwan; ^2^College of Medicine, Chang Gung University, Taoyuan, Taiwan; ^3^Department of Surgery, Chang Gung Memorial Hospital, Taoyuan, Taiwan; ^4^Clinical Informatics and Medical Statistics Research Center and Graduate Institute of Clinical Medicine, Chang Gung University, Taoyuan, Taiwan; ^5^Department of Anesthesiology, Xiamen Chang Gung Hospital, Xiamen, China

## Abstract

**Background:**

Cholecystectomy is one of the most common surgical procedures performed worldwide. The objective of this large, population-based cohort study is to explore the risk factors of pneumonia after cholecystectomy in Taiwan.

**Methods:**

From the Taiwanese National Health Insurance Research Database, we selected all patients who underwent cholecystectomy by using ICD-9-codes, from January 1, 1998, to December 31, 2016. The patients were separated into two groups based on the presence or absence of postoperative pneumonia. Basic information, comorbidities, and postoperative complications were evaluated using a *t*-test or chi-square test. There were 6056 patients in the pneumonia group and 24224 patients in the control group. These two groups were shown in a ratio of 1 : 4 and were matched by age and sex. The log-rank test was used to examine differences in postoperative mortality between patients with and without pneumonia. Preoperative risk factors were analyzed using logistic regression analysis, after adjusting for age and sex.

**Results:**

The final dataset included 282184 cholecystectomy patients. Of these patients, 6056 (2.15%) had postoperative new-onset pneumonia. The patient-related risk factors for pneumonia after cholecystectomy in the order of relevance were chronic obstructive pulmonary disease, congestive heart failure, cerebrovascular disease, diabetes mellitus, surgical type, hemodialysis, coronary artery disease, and liver cirrhosis. Compared to patients without postcholecystectomy pneumonia, those with postcholecystectomy pneumonia had higher rates of mortality (within first month, 1.72% vs. 2.28%, *P* < 0.005) and admission to intensive care unit (15.02% vs. 41.80%, *P* < 0.0001) and longer hospital stays (10.71 vs. 18.55 days, *P* < 0.0001).

**Conclusion:**

Our results show that postcholecystectomy associated with pneumonia had higher rates of morbidity and mortality in this clinical population. Early identification and possible management of risk factors for pneumonia could improve outcomes of cholecystectomy and lower the risk for patient comorbidities after surgery.

## 1. Introduction

Cholecystectomy is one of the most common surgical procedures performed in the world. Generally, it is a safe procedure with low morbidity and mortality rates [1] except in certain higher-risk groups such as older patients, those with cirrhosis, undergoing dialysis, and obese patients [2–5]. The possible postoperative complications include bile leak, bile duct injury, postoperative ileus, surgical site infection, postoperative pneumonia, deep vein thrombosis, pulmonary embolism, postoperative stroke, and heart failure [2, 4].

Postoperative pneumonia is a common morbidity after upper abdominal surgery and is sometimes the leading cause of mortality [6]. Postoperative pneumonia can lead to prolonged hospitalization and increased medical cost. Pulmonary dysfunction is a major complication after upper abdominal surgery. Impaired gas exchange can occur during general anesthesia and can be related to the effects of general anesthesia on change of ventilation, lung and chest wall mechanics, and ventilation-perfusion relationships [7]. Two factors, other than the effect of general anesthesia, lead to postoperative restrictive pulmonary dysfunction. Impaired diaphragmatic function after upper abdominal surgery leads to limited functional residual capacity. Postoperative pain limits the movement of the abdominal wall and results in a shallow respiratory pattern [7, 8]. Overall, pulmonary dysfunction may result in atelectasis, hypoxemia, and pneumonia [9].

In previous literature, although some reports showed the risk factors of postoperative pulmonary complications after major surgery [10], only few studies aimed at the predictions of postoperative pneumonia specifically for cholecystectomy [11, 12]. Our data provided by the National Health Insurance Bureau ensure nationwide coverage; therefore, we believe that this population-based cohort study can help researchers answer questions about low-incidence conditions and generalize the findings to a national population.

The purpose of our study was to estimate the incidence rates of postoperative pneumonia in a cohort of patients undergoing cholecystectomy in Taiwan. We also evaluated the association between postoperative pneumonia and cholecystectomy with respect to demographics, comorbidities, and postpneumonia outcomes. To improve clinical practices, we further analyzed the patient-related risk factors of postoperative pneumonia in patients receiving cholecystectomy. It is important to establish predictors of perioperative pneumonia to ensure that patients and clinicians have the necessary information to make decisions or to arrange for postoperative ICU care.

## 2. Materials and Methods

### 2.1. Data Collection

This was a retrospective, population-based cohort study based on Taiwan's National Health Insurance Research Database (NHIRD). Deidentified computerized data were derived from the Bureau of National Health Insurance (NHI), which organizes claim data for the entire NHI system and has established the NHIRD. The NHI database provides information of over 99% of the Taiwanese population. This database comprises basic patient information, medical data, dates of admission, operations, and clinical diagnostic codes from the International Classification of Disease, Revision 9, Clinical Modification (ICD-9-CM). This study was approved by the Institutional Review Board of Chang Gung Memory Hospital (No.: 202000117B1).

### 2.2. Patient Definition and Selection

**Figure 1:**
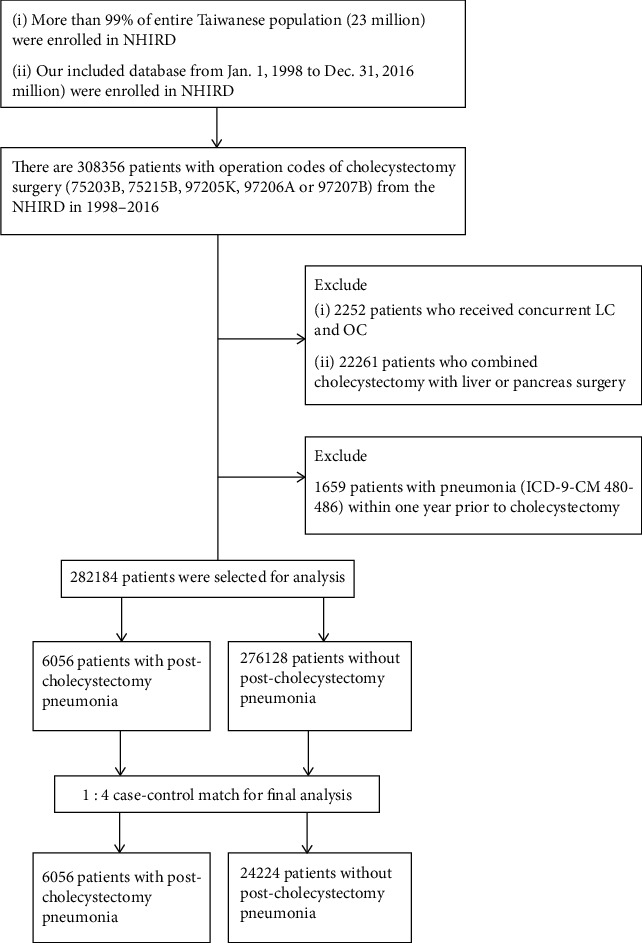
Study design and flow chart of patient selection.

Figure 1 presents the flowchart of patient selection and identification process for our study. From the NHIRD, we identified adult patients (>20 years old) who underwent cholecystectomy between January 1998 and December 2016, using the ICD-9-CM codes. The ICD-9-CM procedure code 51.2 (cholecystectomy) was utilized for identification. ICD-9-CM operation codes of cholecystectomy (75203B) and laparoscopic cholecystectomy (LC) (75215B, 97205K, 97206A, or 97207B) were applied to recognize cholecystectomy. During this period, a total of 308356 patients who underwent cholecystectomy were identified from this database. We excluded 2252 patients who received concurrent LC and open cholecystectomy (OC). The 22261 patients who had additionally undergone liver or pancreatic surgery (ICD-9-CM 50, 52) were excluded. Further, those patients with pneumonia (ICD-9-CM 480-486) diagnosed within one year prior to cholecystectomy were also excluded (*n* = 1659). Finally, the study cohort comprised 282184 patients (Figure 1).

Medical comorbidities were defined as five recorded outpatient department (OPD) diagnoses, one recorded outpatient with one recorded inpatient diagnoses, or two recorded inpatient department (IPD) diagnoses within 24 months before surgery. All diagnoses were verified with the ICD-9-CM codes. Further, the following comorbidities were defined using these codes: hypertension (ICD-9-CM 401-405); diabetes mellitus (ICD-9-CM 250); coronary artery disease (ICD-9-CM 410-414); cerebrovascular disease (ICD-9-CM 430-438); chronic obstructive pulmonary disease (ICD-9-CM 490–492 and 496); hemodialysis (ICD-9-CM procedure codes 58029C, 58001C, and 58027C); liver cirrhosis (ICD-9-CM 571.5); obesity (ICD-9-CM 278); and congestive heart failure (ICD-9-CM 428). New-onset postoperative pneumonia was identified from the relevant ICD-9-CM codes (ICD-9-CM 480-486) for patients who received cholecystectomy within one month. Death was defined as the termination of national health insurance or receipt of insurance death codes.

### 2.3. Measurements

Our primary outcome was the independent risk factors for postcholecystectomy pneumonia, including demographic and clinical risk factors. The secondary outcome was adverse effects of postoperation, including total length of hospital stay, admission rate of ICU, and mortality. Postoperative mortality rates at the first month, second month, and third month were calculated. Primary and secondary outcomes were compared between patients with and without pneumonia.

### 2.4. Statistical Analysis

Between-group differences in the distribution of demographic data, coexisting medical diseases, length of hospitalization, and rates of ICU admission were estimated using a *t*-test or chi-square test, as appropriate for the type and distribution of the data. There were 6056 patients in the pneumonia group and 24224 patients in the control group. These two groups were shown in a ratio of 1 : 4 and were matched by age and sex. The log-rank test was used to examine differences in postoperative mortality between patients with and without pneumonia, and further used to analyze differences between LC and OC. Risk factors for cholecystectomy pneumonia were evaluated using multivariate logistic regression analysis adjusted by age and sex, and individually for preexisting chronic obstructive pulmonary disease, congestive heart failure, cerebrovascular disease, diabetes mellitus, OC (reference group LC), hemodialysis, coronary artery disease, and liver cirrhosis. Multivariate results are reported as odds ratios (ORs) and 95% confidence intervals (CIs). All analyses were performed using SAS software (version 9.3, SAS Institute Inc., Cary, NC), with a two-sided *P* value < 0.05 considered to indicate statistically significant differences.

## 3. Results

### 3.1. Study Population and Characteristics

Between January 1998 and December 2016, a total of 282184 patients who underwent cholecystectomy were included in this study. Overall, 90930 and 191254 patients underwent single OC and LC, respectively. Relevant demographic data and clinical characteristics are shown in Table 1. Overall, 6056 (2.15%) patients had postoperative new-onset pneumonia (Table 1). The incidence rate of postoperative pneumonia was higher among patients undergoing OC than LC (2.85% vs. 1.81%). In general, patients with postoperative pneumonia tended to be older, male, and receiving OC. Because of the significant heterogeneity of patient number among the demographics of the two groups, cohorts were created and matched in a 1 : 4 ratio of patient : control based on age and sex (Table 2).

Cholecystectomy patients with postoperative pneumonia were more likely to have a higher risk of preoperative diabetes mellitus (26.21% vs. 18.68%, *P* < 0.0001); coronary artery disease (17.92% vs. 12.47%, *P* < 0.0001); cerebrovascular diseases (15.08% vs. 9.32%, *P* < 0.0001); chronic obstructive pulmonary disease (24.79% vs. 7.12%, *P* < 0.0001); hemodialysis (4.26% vs. 2.94%, *P* < 0.0001); liver cirrhosis (3.22% vs. 1.49%, *P* < 0.0001); and congestive heart failure (2.06% vs. 0.99%, *P* < 0.0001) than those without postoperative pneumonia.

### 3.2. Predictors of Postcholecystectomy Pneumonia

Multivariable logistic regression analysis was performed to determine independent predictors of postoperative pneumonia after cholecystectomy (Table 3). All risk factors identified in Table 2 were examined. The odds of postoperative pneumonia in the order of relevance were chronic obstructive pulmonary disease, congestive heart failure, cerebrovascular disease, diabetes mellitus, OC, hemodialysis, coronary artery disease, and liver cirrhosis.

### 3.3. Postpneumonia Outcomes

Clinical outcomes identified by univariate analysis as being associated with postcholecystectomy pneumonia are reported in Table 4. The duration of hospitalization was longer in cholecystectomy patients with pneumonia than those without pneumonia (*P* < 0.0001). The incidence rate of postoperative ICU admission was higher in the group with pneumonia than that without (Table 4). The most notable finding was the ICU admission rate of the pneumonia group was as high as 41.80%. Furthermore, the postcholecystectomy mortality rate of patients with pneumonia was also higher than that of patients without pneumonia (Table 5), especially during the first month after surgery (2.28%), though it declined over time. There was no difference between these two groups in the third month.

In addition, for cholecystectomy patients with postoperative pneumonia, the mortality rate of OC was higher than that of LC during the first three months after surgery (9.56% vs. 1.01%, *P* < 0.0001) (Table 6). All differences in outcomes reached statistical significance.

## 4. Discussion

Postoperative pneumonia is one of the most important causes of postoperative morbidity and mortality after abdominal surgery [13]. There is very limited data available regarding determination of predictors of postoperative pneumonia in a large sample of cholecystectomy patients. We performed a retrospective, population-based cohort study of patients who underwent cholecystectomy between 1998 and 2016, with the aim of describing the incidence, risk factors, and outcomes associated with postcholecystectomy pneumonia.

We found that 2.15% patients receiving cholecystectomy in Taiwan developed postoperative pneumonia, with 1.81% and 2.85% belonging to the LC and OC groups, respectively. In a previous observational study, the incidence rates of pneumonia after cholecystectomy were 0.8% and 3.6% for the LC and OC groups, which were similar to our study outcomes [14].

In this study, we determined that preoperative factors are associated with an increased risk of postoperative pneumonia. Their odds ratios by multivariate regression analysis range approximately between 1.00 and 4.0. The first independent risk factor of postcholecystectomy pneumonia was preoperative chronic obstructive pulmonary disease, which is the most significant predictor in our study. Several studies have examined the association between chronic obstructive pulmonary disease and postoperative pneumonia [13, 15, 16]. Results about whether chronic obstructive pulmonary disease is correlated with postoperative pulmonary complications are controversial. A retrospective cohort study with 387 patients showed that mild-to-moderate chronic obstructive pulmonary disease may not be a significant risk factor for pneumonia after abdominal surgery [13]. However, this study had a small sample size and not only for patients receiving cholecystectomy. Liao et al. [16] reported a larger retrospective study wherein patients with chronic obstructive pulmonary disease had a higher risk for postoperative pulmonary complications after LC than those without chronic obstructive pulmonary disease. This result is similar to ours. In our report, the odds ratio was 4.14 for postoperative pneumonia among those with chronic obstructive pulmonary disease compared to those without. Patients with chronic pulmonary disease were more susceptible to pneumonia because of the underlying destruction of lung structure [16].

The second independent predictor was congestive heart failure. Previous studies showed that pneumonia risk was associated with the degree of ventricular function impairment, and heart failure was an independent risk factor for pneumonia in general population [17]. Hall et al. performed a large retrospective study which identified decompensated congestive heart failure to be the predictor of postoperative respiratory failure in LC [11]. In our study, we found that a close association between congestive heart failure and postoperative pneumonia was identified in cholecystectomy patients.

Cerebrovascular disease is a previously less-mentioned risk factor associated with postoperative pulmonary complication [18, 19]. One study found an association between cerebrovascular accident and postoperative pneumonia after noncardiac major surgery [18]. Our study also showed that cerebrovascular accident is closely associated with postoperative pneumonia after cholecystectomy.

Our study found that surgical procedure is another risk factor for the development of postcholecystectomy pneumonia. In previous literature, the laparoscopic approach for abdominal surgery resulted in fewer postoperative pulmonary complications than open procedures [20, 21]. Furthermore, Lee et al. found that patients who received an open right hemicolectomy were 1.78 times more likely to be hospitalized for pneumonia than those who received a laparoscopic right hemicolectomy [22]. In our study, the patients receiving OC had a 1.59 times higher risk for pneumonia than those receiving LC. Previous studies have shown that pulmonary function after LC was better than that after OC [9, 23, 24]. Postoperative pulmonary dysfunction has primarily been considered a restrictive process. These changes may lead to the development of atelectasis, hypoxemia, and pneumonia [9].

The other independent risk factors for postoperative pneumonia identified in our study were diabetes mellitus, hemodialysis, liver cirrhosis, and coronary artery disease. The first three factors had been mentioned in previous studies [25–27]. Coronary artery disease was also found to be an independent risk factor for pneumonia in general population who did not undergo surgery [17]. Consistent with our findings, the higher risk of pneumonia was associated with coronary artery disease.

Moreover, in our study, obesity was not associated with postcholecystectomy pneumonia. Previous study had reported that obesity is the predictor of postoperative pulmonary complications following abdominal surgery [28]. However, the sample size of that study was relatively small, which limited the number of potential risk factors that could be evaluated.

In this study, we found out that chronic obstructive pulmonary disease, cerebrovascular disease, diabetes mellitus, hemodialysis, liver cirrhosis, and heart disease are the important risk factors of postcholecystectomy pneumonia. The improvement in the treatment of comorbidities can reduce the incidence of pneumonia and complications after cholecystectomy.

Our large retrospective population-based cohort study has some limitations. First, we could not recognize the severity of preoperative medical comorbidities, as the severity is not classified with codes. Second, NHIRD is a secondary database and therefore does not include detailed medical data such as intraoperative data, hemodynamics, operative time, and medications used during the surgery that may be closely linked with the development of postoperative pneumonia. Third, our cohort included patients over an 18-year period (1998–2016). In Taiwan, LC was first performed in the 1990s [29], and laparoscopic procedure was significantly growing in recent years. However, our data was collected from 1998 and the average percentage of OC showed a higher proportion than the current recommended standard. In addition, the treatment for comorbidities has significantly improved over this period of time. However, despite these limitations, the data provided by the NHI Bureau are generally accurate and provide nationwide coverage. Therefore, our study may help researchers answer questions about low-incidence conditions and generalize findings to a national population.

## 5. Conclusion

The major strength of our study is the large sample size selected from a population-based database rather than a single institution, providing an adequate representation of the risk factors of pneumonia following cholecystectomy. Our results show that postcholecystectomy associated with pneumonia had higher rates of ICU admission and mortality in this clinical population. The predictors for pneumonia after cholecystectomy in the order of relevance were chronic obstructive pulmonary disease, congestive heart failure, cerebrovascular disease, surgical type, diabetes mellitus, hemodialysis, coronary artery disease, and liver cirrhosis. Early identification and advances in treatment of these risk factors could improve the short- and long-term outcomes for postcholecystectomy pneumonia. Besides, the patients receiving OC had a higher risk for pneumonia than those receiving LC. Our population-based study in Taiwan provides additional evidence that the increasing trends toward LC could benefit the outcomes of patients.

## Figures and Tables

**Table 1:** General demographics of the study subjects.

Variable	Non-postop pneumonia (*n* = 276128)	Postop pneumonia (*n* = 6056)	*P* value
Age			<0.0001
Mean (SD)	56.52 (15.98)	65.84 (15.53)	
Sex			<0.0001
Female	145566 (52.72%)	2902 (47.92%)	
Male	130562 (47.28%)	3154 (52.08%)	
Surgical type			<0.0001
OC	88337 (31.99%)	2593 (42.82%)	
LC	187791 (68.01%)	3463 (57.18%)	
Preoperative clinical parameter			
Hypertension	56134 (20.33%)	1804 (29.79%)	<0.0001
DM	39845 (14.43%)	1587 (26.21%)	<0.0001
CAD	30922 (11.20%)	1085 (17.92%)	<0.0001
CVA	15923 (5.77%)	913 (15.08%)	<0.0001
COPD	13922 (5.04%)	1501 (24.79%)	<0.0001
Hemodialysis	5557 (2.01%)	258 (4.26%)	<0.0001
Liver cirrhosis	2963 (1.07%)	195 (3.22%)	<0.0001
Obesity	2702 (0.98%)	44 (0.73%)	0.0562
CHF	2416 (0.87%)	125 (2.06%)	<0.0001

A *t*-test or chi-square test was used to examine the differences in the demographic characteristics of cholecystectomy patients. OC: open cholecystectomy; LC: laparoscopic cholecystectomy; DM: diabetes mellitus; CAD: coronary artery disease; CVA: cerebrovascular disease; COPD: chronic obstructive pulmonary disease; CHF: congestive heart failure.

**Table 2:** 1 : 4 case-control match of the risk factors based on age and sex.

Variable	Non-postop pneumonia (*n* = 24224)	Postop pneumonia (*n* = 6056)	*P* value
Age			0.4687
Mean (SD)	65.68 (15.39)	65.84 (15.53)	
Sex			1.0000
Female	11608 (47.92%)	2902 (47.92%)	
Male	12616 (52.08%)	3154 (52.08%)	
Surgical type			<0.0001
OC	10025 (41.38%)	2593 (42.82%)	
LC	14199 (58.62%)	3463 (57.18%)	
Preoperative clinical parameter			
Hypertension	6940 (28.65%)	1804 (29.79%)	0.4557
DM	4526 (18.68%)	1587 (26.21%)	<0.0001
CAD	3021 (12.47%)	1085 (17.92%)	<0.0001
CVA	2258 (9.32%)	913 (15.08%)	<0.0001
COPD	1724 (7.12%)	1501 (24.79%)	<0.0001
Hemodialysis	711 (2.94%)	258 (4.26%)	<0.0001
Liver cirrhosis	361 (1.49%)	195 (3.22%)	<0.0001
Obesity	167 (0.69%)	44 (0.73%)	0.7559
CHF	241 (0.99%)	125 (2.06%)	<0.0001

A *t*-test or chi-square test was used to examine the differences in the demographic characteristics of the 1 : 4 case-control match among cholecystectomy patients based on age and sex. OC: open cholecystectomy; LC: laparoscopic cholecystectomy; DM: diabetes mellitus; CAD: coronary artery disease; CVA: cerebrovascular disease; COPD: chronic obstructive pulmonary disease; CHF: congestive heart failure.

**Table 3:** Predictors of pneumonia after cholecystectomy by multivariate analysis.

Parameter	Odds ratio	95% CI	*P* value
COPD	4.147	(3.845-4.474)	<0.0001
CHF	1.965	(1.587-2.438)	<0.0001
CVA	1.685	(1.552-1.830)	<0.0001
DM	1.661	(1.555-1.774)	<0.0001
OC (ref. group LC)	1.596	(1.512-1.676)	<0.0001
Hemodialysis	1.475	(1.263-1.722)	<0.0001
CAD	1.354	(1.251-1.467)	<0.0001
Liver cirrhosis	1.167	(1.017-1.337)	0.0002

Multiple logistic regression was used to examine the odds ratio individually adjusted by age and sex for the risk factors. CI: confidence interval; COPD: chronic obstructive pulmonary disease; CHF: congestive heart failure; CVA: cerebrovascular disease; DM: diabetes mellitus; OC: open cholecystectomy; LC: laparoscopic cholecystectomy; ref.: reference; CAD: coronary artery disease.

**Table 4:** Outcome characteristics of patients after cholecystectomy with or without pneumonia.

	Without pneumonia (*n* = 24224)	With pneumonia (*n* = 6056)	*P* value
	Mean (SD)/*n* (%)	Mean (SD)/*n* (%)	
Hospital stay (days)	10.71 (11.82)	18.55 (15.30)	<0.0001
ICU admission rate	3639 (15.02%)	2532 (41.80%)	<0.0001

Continuous variables were described as the mean ± standard deviation (SD) and the categorical variable as number of event (*n*/%). ICU: intensive care unit; SD: standard deviation.

**Table 5:** Mortality rates of patients after cholecystectomy with or without pneumonia.

	Without pneumonia (*n* = 24224)	With pneumonia (*n* = 6056)	*P* value
	*n* (%)	*n* (%)	
Mortality (1 month)	416 (1.72%)	138 (2.28%)	0.0035
Mortality (2 months)	276 (1.14%)	92 (1.52%)	0.0158
Mortality (3 months)	157 (0.65%)	53 (0.88%)	0.0569

The log-rank test was used to examine the differences in the mortality rates of the 1 : 4 case-control match after cholecystectomy.

**Table 6:** Mortality rates of patients with pneumonia after LC or OC.

	With pneumonia (*n* = 6056)				*P* value
	LC (*n* = 3463)		OC (*n* = 2593)		
	*n*	%	*n*	%	
Mortality (1 month)	19	0.55	119	4.58	<0.0001
Mortality (2 months)	9	0.26	83	3.20	<0.0001
Mortality (3 months)	7	0.20	46	1.77	<0.0001
Mortality (≤3 months)	35	1.01	248	9.56	<0.0001

The log-rank test was used to examine the differences in mortality rates of postcholecystectomy patients with pneumonia. OC: open cholecystectomy; LC: laparoscopic cholecystectomy.

## Data Availability

The data used to support the findings of this study are available from the corresponding author upon request.
